# Detecting oxidants in muscle *in vivo*: A perspective on “State of the art *in vivo* reactive oxygen species measurements in skeletal muscle using fluorescent probes”

**DOI:** 10.1016/j.rbc.2026.100073

**Published:** 2026-03

**Authors:** Malcolm J. Jackson

**Affiliations:** Department of Musculoskeletal and Ageing Science, Institute of Life Course and Medical Sciences, University of Liverpool, L7 8TX, UK

The recent review by Kano et al. [1] provides an excellent overview of state-of-the-art approaches to monitor reactive oxygen species (ROS) in skeletal muscle at rest and during contractile activity. The authors have pioneered the use of advanced HyPer7 probes to monitor changes in hydrogen peroxide (H_2_O_2_) within distinct subcellular compartments *in vivo* [2,3] and in this review they highlight striking discrepancies between the magnitude of ROS increases observed *in vivo* (1.06-1.1 fold) and those reported in *in vitro* preparations (1.0-4 fold). They attribute this difference primarily to the elevated intracellular oxygen tensions typical of *in vitro* systems, concluding that “the elevated ROS levels reflect hyperoxic dysregulation rather than physiological responses to contractions per se.”

While this explanation is compelling, additional factors in experimental design are also likely to have contributed to these discrepancies. It is suggested that differences in (1) the nature of the ROS-sensitive probes employed, (2) the partial pressure of oxygen (pO_2_) in the muscle preparations, and (3) the presence or absence of an intact circulation are all likely contributors to the apparent discrepancies.

## 1 Nature of the ROS-sensitive probes

Kano et al. [1] emphasize the superior specificity of HyPer7 for H_2_O_2_ compared with traditional, less selective probes such as DCFH and DHE. Given HyPer7's high selectivity toward H_2_O_2_ [4], it is perhaps unsurprising that the fold-change detected during contractile activity differs from that observed with probes that react with multiple ROS and other oxidants [5]. Although no studies have yet employed HyPer7 in *in vitro* systems, experiments using HyPer2 in isolated muscle fibres showed no significant increase during contractions, but a delayed post-contraction rise [6]. Similarly, studies using another H_2_O_2_-specific probe, roGFP2-Orp1, reported only modest (∼1.3-fold) cytosolic increases during contraction [7]. These findings suggest that the high specificity of HyPer7 may itself underlie part of the observed *in vivo*/*in vitro* divergence. A direct comparison of HyPer7 oxidation in both experimental contexts would be valuable.

## 2 Partial pressure of oxygen in the muscle

Traditional *in vitro* preparations often employ oxygenation with 95% O_2_/5% CO_2_ to maintain tissue viability, as in early *ex vivo* studies of rat diaphragm [8]. The review authors point out that this approach likely elevates intracellular pO_2_ to >150 Torr, compared with *in vivo* muscle values of 2–30 Torr [1]. The majority of the *in vitro* studies cited have not used this approach with more recent examining myotubes or isolated mouse flexor digitorum brevis (FDB) fibres cultured under 20% O_2_/5% CO_2_, this achieves lower, but still supra-physiological pO_2_ values [6,9,10]. It is notable that even this moderate elevation has been reported to influence ROS production since increasing ambient O_2_ from 6% to 20% enhanced DCFH and DHE oxidation by ∼30% in muscle myotubes at rest [11]. Thus, while differences in oxygenation likely contribute to higher ROS signals *in vitro*, the magnitude of this effect alone may not fully account for the observed discrepancies.

## 3 Presence of an intact blood circulation

Kano et al. also suggest that the intact circulation in *in vivo* experiments may attenuate observed HyPer7 signals because H_2_O_2_ generated by myocytes could be rapidly released into the bloodstream. However, this seems unlikely. Cytosolic H_2_O_2_ concentrations are typically in the 5–10 nM range, with a substantial gradient across the plasma membrane with extracellular levels calculated to be approximately 650 fold higher [12] making passive diffusion improbable. Instead, contraction-induced ROS formation via Nox2 likely occurs at the extracellular face of the sarcolemma, generating H_2_O_2_ in the interstitial space rather than directly into the circulation. Given that interstitial fluid flow (0.1–2.0 μm/s) is orders of magnitude slower than blood flow (100–200 cm/s), locally generated H_2_O_2_ is unlikely to be rapidly removed. Rather, degradation of generated H_2_O_2_ by extracellular antioxidants such as glutathione peroxidase may limit local interstitial H_2_O_2_ accumulation *in vivo*. This buffering would not occur in isolated fibre systems, where the extracellular environment is simplified, possibly amplifying observed ROS signals.

## 4 Concluding remarks

The review by Kano et al. provides valuable insight into *in vivo* ROS measurement in skeletal muscle and highlights key methodological considerations. The additional reflections provided here suggest that differences in probe specificity, oxygen tension, and the extracellular milieu may all contribute to the observed disparities between *in vivo* and *in vitro* findings. Addressing these factors will further advance our understanding of the mechanisms governing H_2_O_2_ and other ROS generation in contracting skeletal muscle.

## Declaration of competing interest

The author declares that they have no known competing financial interests or personal relationships that could have appeared to influence the work reported in this paper.

## References

[1] Kano R., Shirakawa H., Poole D.C., Hoshino D., Kano Y. (2025). State of the art in vivo reactive oxygen species measurements in skeletal muscle using fluorescent proteins. Redox Biochem. Chem..

[2] Kano R., Kusano T., Takeda R., Shirakawa H., Poole D.C., Kano Y., Hoshino D. (2024). Eccentric contraction increases hydrogen peroxide levels and alters gene expression through Nox2 in skeletal muscle of male mice. J. Appl. Physiol..

[3] Kano R., Tabuchi A., Tanaka Y., Shirakawa H., Hoshino D., Poole D.C., Kano Y. (2024). In vivo cytosolic H(2)O(2) changes and Ca(2+) homeostasis in mouse skeletal muscle. Am. J. Physiol. Regul. Integr. Comp. Physiol..

[4] Pak V.V., Ezerina D., Lyublinskaya O.G., Pedre B., Tyurin-Kuzmin P.A., Mishina N.M., Thauvin M., Young D., Wahni K., Martinez Gache S.A., Demidovich A.D., Ermakova Y.G., Maslova Y.D., Shokhina A.G., Eroglu E., Bilan D.S., Bogeski I., Michel T., Vriz S., Messens J., Belousov V.V. (2020). Ultrasensitive genetically encoded indicator for hydrogen peroxide identifies roles for the oxidant in cell migration and mitochondrial function. Cell Metab..

[5] Kalyanaraman B., Darley-Usmar V., Davies K.J., Dennery P.A., Forman H.J., Grisham M.B., Mann G.E., Moore K., Roberts L.J., Ischiropoulos H. (2012). Measuring reactive oxygen and nitrogen species with fluorescent probes: challenges and limitations. Free Radic. Biol. Med..

[6] Pearson T., Kabayo T., Ng R., Chamberlain J., McArdle A., Jackson M.J. (2014). Skeletal muscle contractions induce acute changes in cytosolic superoxide, but slower responses in mitochondrial superoxide and cellular hydrogen peroxide. PLoS One.

[7] Henríquez-Olguin C., Knudsen J.R., Raun S.H., Li Z., Dalbram E., Treebak J.T., Sylow L., Holmdahl R., Richter E.A., Jaimovich E., Jensen T.E. (2019). Cytosolic ROS production by NADPH oxidase 2 regulates muscle glucose uptake during exercise. Nat. Commun..

[8] Reid M.B., Haack K.E., Franchek K.M., Valberg P.A., Kobzik L., West M.S. (1992). Reactive oxygen in skeletal muscle. I. Intracellular oxidant kinetics and fatigue in vitro. J. Appl. Physiol..

[9] Palomero J., Pye D., Kabayo T., Spiller D.G., Jackson M.J. (2008). In situ detection and measurement of intracellular reactive oxygen species in single isolated mature skeletal muscle fibers by real time fluorescence microscopy. Antioxidants Redox Signal..

[10] Sakellariou G.K., Vasilaki A., Palomero J., Kayani A., Zibrik L., McArdle A., Jackson M.J. (2013). Studies of mitochondrial and nonmitochondrial sources implicate nicotinamide adenine dinucleotide phosphate oxidase(s) in the increased skeletal muscle superoxide generation that occurs during contractile activity. Antioxidants Redox Signal..

[11] McCormick R., Pearson T., Vasilaki A. (2016). Manipulation of environmental oxygen modifies reactive oxygen and nitrogen species generation during myogenesis. Redox Biol..

[12] Huang B.K., Sikes H.D. (2014). Quantifying intracellular hydrogen peroxide perturbations in terms of concentration. Redox Biol..

